# Dual E-Cigarette Users Show Nicotine Addiction Risk Alleles and Nuclear Abnormalities in Oral Epithelial Cells

**DOI:** 10.3390/arm94030039

**Published:** 2026-06-18

**Authors:** Oreth Montero-Ruiz, Ramcés Falfán-Valencia, Ivette Buendía-Roldán, Daniela Valencia-Pérez Rea, Gibran E. Rueda-Munive, Ingrid Fricke-Galindo, Salvador García-Carmona, Edgar Abarca-Rojano, Gloria Pérez-Rubio

**Affiliations:** 1 Laboratory of Biochemistry and Neurotoxicology, Faculty of Bioanalysis-Xalapa, Universidad Veracruzana, Médicos y Odontólogos S/N Unidad del Bosque, Xalapa 91010, Mexico; 2 Pneumogenomics Laboratory, Instituto Nacional de Enfermedades Respiratorias Ismael Cosío Villegas, Mexico City 14080, Mexico; 3 Translational Research Clinic on Aging and Pulmonary Fibrosis, Instituto Nacional de Enfermedades Respiratorias Ismael Cosio Villegas, Mexico City 14080, Mexico; 4 Sección de Posgrado e Investigación, Escuela Superior de Medicina, Instituto Politécnico Nacional, Mexico City 11340, Mexico

**Keywords:** micronuclei, genetic, dual user, e-cigarette, smoking, combustible cigarette

## Abstract

**Highlights:**

**What are the main findings?**

- In dual e-cigarette users, carrying the A (rs16969968), C (rs1800955), and T (rs4105144) alleles in the *CHRNA5*, *DRD4*, and *CYP2A6* genes, respectively, is associated with traits linked to increased nicotine dependence.
- Nicotine use, whether from electronic or combustible cigarettes, increases the number of micronuclei and other nuclear abnormalities in oral epithelial cells.

**What are the implications of the main findings?**

- Dual e-cigarette users carry alleles in genes linked to traits associated with higher nicotine addiction, raising the risk of tobacco smoking and related diseases.
- Dual use of electronic cigarettes increases the frequency of micronuclei and nuclear abnormalities in oral epithelial cells, effects comparable to those observed in users of combustible cigarettes.

**Abstract:**

Background: This study was conducted to identify genetic risk variants associated with nicotine addiction in the *CHRNA5*, *HTR2A*, *DRD4*, and *CYP2A6* genes among electronic cigarette users who also smoke combustible cigarettes (dual users), and to assess potential genotoxic and cytotoxic damage in the oral mucosal cells of the study population. Methods: We included dual e-cig users (ECIG, *n* = 70), combustible cigarette smokers (CCU, *n* = 24), and non-smokers and non-e-cig users (NS, *n* = 110). Genetic variants in *CHRNA5*, *HTR2A*, *DRD4*, and *CYP2A6* were genotyped. Micronucleus analysis was performed on oral mucosal cells to detect cellular abnormalities. Results: The ECIG group demonstrated greater nicotine addiction on the Fagerström Test for Nicotine Dependence (FTND, 5.5 vs. 1, *p* = 0.023). Salivary cotinine levels were significantly higher in the ECIG group compared to the CCU group (39 vs. 12 ng/mL, *p* < 0.001). The carriers of the A allele (rs16969968/*CHRNA5*) had higher FTND scores, carriers of the C allele (rs1800955/*DRD4*) used electronic cigarettes more frequently each day, and carriers of the T allele (rs4105144/*CYP2A6*) started using nicotine products at a younger age. The number of micronuclei and cellular abnormalities in the oral mucosa was higher in the ECIG and CCU groups compared to the NS group. Conclusions: Salivary cotinine levels and FTND are higher in dual e-cigarette users than in combustible cigarette users. Dual users exhibit risk alleles in the *CHRNA5*, *DRD4*, and *CYP2A6* genes, which are associated with traits linked to increased nicotine addiction. Dual e-cigarette use poses comparable genotoxic risks to combustible smoking.

## 1. Introduction

The World Health Organization (WHO) considers the persistent marketing of electronic cigarettes (e-cigs) to young people a cause for concern [1]. In Mexico, the sale, manufacture, distribution, and marketing of these products are prohibited [2]; however, they can be purchased online and in local stores. Data from 2022 showed that 10% of Mexican adolescents use e-cigs; only 4% are exclusive e-cig users [3]. In Mexico, as in other Caucasian populations, dual use is more common: nearly half of e-cigarette users also smoke combustible cigarettes [4]. Several studies have found that higher use of e-cigs is linked to a three to four times greater likelihood of using combustible cigarettes [5].

The nicotine content of early e-cigarettes ranged from 3 to 36 mg/mL, while the most recent generations contain up to 80 mg/mL. Manufacturers use nicotine in salt form to lower the pH, improve alkaloid absorption, and reduce inhalation irritation [6].

The mechanism of addiction to the nicotine contained in e-cigs is the same as that observed in combustible cigarettes; however, in dual users, the level of addiction is higher than in those who use only e-cigs [7]. It is now clear that substance addiction is a complex and multifactorial disease influenced by genetics and the environment [8].

When nicotine enters the body, it is metabolized in the liver, primarily by the cytochrome P450 2A6 enzyme, which converts it to cotinine. The gene encoding this enzyme, *CYP2A6*, is highly polymorphic, and genetic variants lead to differences in the nicotine metabolite profile [9,10]. Common alleles with no or intermediate activity have been identified, as well as less frequent alleles with uncertain activity. Of particular interest, the rs1137115 variant reduces mRNA splicing efficiency, resulting in slower in vivo nicotine metabolism [11]. In contrast, the rs4105144 variant is associated with alleles that encode loss-of-function enzymes, thereby further diminishing enzymatic activity [12]. Thus, the presence of these risk alleles in *CYP2A6* decreases the rate at which nicotine is metabolized. Nicotine binds to nicotinic cholinergic receptors in the central nervous system, stimulating the release of dopamine, glutamate, and serotonin [13]. Chronic nicotine intake raises dopaminergic activity, leading to compulsive cravings, and also affects the serotonergic system, which contributes to nicotine’s pleasurable effects [10].

Several studies have identified single-nucleotide polymorphisms (SNPs) associated with nicotine addiction, early initiation of cigarette use, and nicotine metabolism rate. Among them, rs16969968, located in *CHRNA5* (cholinergic receptor nicotinic alpha 5 subunit), has been associated with an increased risk of nicotine addiction in Caucasian, African American, and Mexican mestizo populations [14]. The rs6311 and rs6313 (*HTR2A*, gene that encodes one of the receptors for serotonin) polymorphisms have been associated with cigarette smoking and the degree of nicotine addiction [15]. In contrast, rs1800955, located in *DRD4* (dopamine receptor D4), has been associated with alterations in dopaminergic neurotransmission, a mechanism that influences the reinforcing and maintenance effects of nicotine use [16]. These genetic variants have also been associated with an increased risk of developing pathologies such as chronic obstructive pulmonary disease and lung cancer [17].

Across different populations, the genetic variant rs16969968 (*CHRNA5*) has demonstrated the most consistently replicated significant association with nicotine addiction or smoking-related traits. The presence of an “A” allele (instead of the more typical “G” allele) causes a change in the protein from aspartate to asparagine at position 398 of the α5 subunit of the nicotinic cholinergic receptor protein [18]. In mouse experiments, the A allele was found to cause partial loss of function at the protein level, requiring increased nicotine intake to activate dopaminergic neurons effectively [19]. Regarding the dopaminergic pathway, the *DRD4* gene contains the rs1800955 polymorphism in its promoter region. The T allele reduces the number of dopamine D4 receptors at the synapse. Thus, it has been suggested that carriers of the C allele express more dopamine D4 receptors, resulting in a greater need for nicotine to stimulate this pathway [20]. Both genetic variants influence the pleasure and reward brain mechanisms involved in nicotine addiction, and this pattern is also observed in individuals who use both regular cigarettes and e-cigarettes (dual users).

Genetic variants in *CYP2A6* are implicated in nicotine metabolism. rs4105144 is a closely related variant of the gene that shows linkage disequilibrium with alleles associated with a slow metabolic rate [21]. The T allele has been associated with an increased risk of cigarette smoking and with the risk of lung cancer and chronic obstructive pulmonary disease [22].

DNA damage has been observed in oral cavity cells of e-cigarette users. The degree of damage depends on the type of device used and the flavor, with sweet and menthol flavors showing the greatest potential for DNA damage [23]. Smoking is well known to be genotoxic and cytotoxic; chronic use produces biological effects that lead to smoking-related diseases [24]. The micronucleus (MN) assay is the most widely used method for assessing genotoxicity. The number of MNs in epithelial border cells of combustible cigarette smokers and e-cigarette users has been reported to be higher than in individuals who have never smoked [25]. This study was conducted to identify genetic risk variants associated with nicotine addiction in the genes *CHRNA5*, *HTR2A*, *DRD4*, and *CYP2A6* among dual users of electronic cigarettes and combustible cigarettes, and to assess genotoxic and cytotoxic damage to the oral mucosa of the studied population.

## 2. Materials and Methods

### 2.1. Study Population

This comparative observational study included two groups of nicotine users: (1) dual e-cig users (ECIG, *n* = 70) and (2) exclusive users of combustible cigarettes (CCU, *n* = 24). Additionally, there was a group comprising non-smokers and non-users of e-cigs (NS, *n* = 110).

This study was conducted in strict compliance with current ethical regulations for research involving human subjects. The principles established in the Declaration of Helsinki were followed. The protocol was approved by the Research Ethics Committee of the Instituto Nacional de Enfermedades Respiratorias Ismael Cosío Villegas (INER) in Mexico City (protocol number B26-20, approved on 23 September 2020) and by the “Sistema de Registro y Evaluación de la Investigación”, Universidad Veracruzana (Number SIREI 473642023120, 2 August 2023). Before sample and data collection, potential participants were invited, and those who accepted provided their written informed consent. Each participant was assigned an alphanumeric code, and the biological samples were processed at the Pneumogenomics Laboratory of the Instituto Nacional de Enfermedades Respiratorias Ismael Cosío Villegas in Mexico City. Each participant underwent a pulmonary function test (spirometry).

We employed non-probabilistic convenience sampling, recruiting participants from universities in Mexico between 2022 and 2025. All participants agreed voluntarily, and only those over 18 years of age were included. Participants in the ECIG group had used e-cigarettes containing nicotine for at least the previous 6 months, and participants in the CCU group were required to have consumed at least one combustible cigarette daily for six months or more. Individuals who had been diagnosed with cardiovascular disease, autoimmune disease, asthma, allergies, liver disease, diabetes, oral disease or infection, hypertension, pregnancy, bronchial hyperreactivity, cancer, or metabolic diseases, or had undergone chemotherapy were excluded, as were users of other drugs such as marijuana, cocaine, or alcohol. The criteria for saliva and blood sample collection were that no food or beverages were ingested, and that no vaping or smoking occurred within 2 h of sample collection.

We used a modified Fagerström Test for Nicotine Dependence (FTND). We used the same questionnaire for the ECIG and CCU groups and changed the word “tobacco” to “electronic cigarette” according to each participant’s consumption. Total scores were used to assess dependence: 0–3 points indicated no dependence, 4–8 low dependence, 9–12 medium dependence, and 13 or more high dependence [26].

### 2.2. Blood Sampling, DNA Extraction, and Genotyping

Peripheral blood samples were collected in EDTA tubes and centrifuged at 4500 rpm for 5 min. The blood cells underwent further processing for DNA extraction. Genomic DNA was isolated using the Blood DNA Preparation Solution Kit (Jena Bioscience, Jena, Germany), following the manufacturer’s instructions. DNA concentration was measured with a NanoDrop 2000 spectrophotometer (Thermo Scientific, Waltham, MA, USA) via UV–visible spectrophotometry. Ratios of 260/280 and 260/230 were used to evaluate sample purity (1.8–2.0 and >2, respectively). Each sample was then adjusted to a concentration of 15 ng/µL for genotyping. Six SNPs in four genes—*CHRNA5* (rs16969968), *HTR2A* (rs6311, rs6313), *DRD4* (rs1800955), and *CYP2A6* (rs1137115, rs4105144)—were genotyped using real-time Polymerase Chain Reaction (PCR) with a Step One Plus Real-Time PCR System (Applied Biosystems, Foster City, CA, USA) through allelic discrimination with predesigned TaqMan probes (Applied Biosystems, Carlsbad, CA, USA) for each variant (Table S1). Reactions were performed in MicroAmp^®^ Optical 96-well plates (Applied Biosystems; Woolston, UK) using 3 µL of the adjusted DNA per sample, according to the manufacturer’s instructions. Four non-template controls served as negative controls; additionally, 5% of the samples were genotyped using a duplicate-like allelic designation control. This technique is considered reliable and is widely employed for genotyping single-nucleotide variants.

### 2.3. Salivary Cotinine Determination

Saliva was collected in a 50 mL polypropylene sterile centrifuge tube (Corning tube, Auburn, AL, USA). The samples were centrifuged at 4500 rpm for 5 min, separated using micropipettes, and stored at −80 °C until analysis. Human cotinine (catalog no. MBS720957; MyBioSource, San Diego, CA, USA; sensitivity 1 ng/mL; detection range 5–100 ng/mL) was measured in the samples (100 µL) using a competitive enzyme-linked immunosorbent assay (ELISA) according to the manufacturer’s protocol. Absorbance was read at 450 nm. Standards and samples were analyzed in duplicate. The mean well value was recorded in ng/mL.

### 2.4. Buccal Cell Collection, Sample Preparation, and Micronucleus Analysis

Participants were asked to rinse their mouths with an alcohol-free mouthwash (Listerine, Cool Mint Mild, Mexico City, Mexico). Buccal cells were collected using a cytological brush by scraping the inner surfaces of both cheeks and transferred to a glass slide. Two slides (one from each cheek) were prepared for each participant. The buccal cells were then fixed to the slides with 95% ethanol for one minute (HYCEL, Jalisco, Mexico). The samples were subsequently processed for histopathological evaluation using Giemsa staining (HYCEL, Jalisco, Mexico, diluted to 10% in Sorensen phosphate buffer pH 7. Table S2) for 10 min. All preparations were examined under a light microscope (Zeiss, Primostar 3, Oberkochen, Baden-Württemberg, Germany) at 10× magnification for detection and 40× magnification for counting cells with MN. For each participant, 1000 cells were evaluated, and a single examiner recorded the frequency of MN. Cells were included in the analysis if their cytoplasm was intact; if the flap cells in the preparation were in the proper position; if there was minimal or no overlap with adjacent cells, minimal or no debris, and a normal, intact nucleus with a smooth, well-defined nuclear perimeter and no overlap of nuclei. Cells with two nuclei, degenerated or dead cells, and cells with an MN-like structure connected to the main nucleus by a bridge were excluded [27].

### 2.5. Statistical Analysis

Data were recorded in a spreadsheet for subsequent analysis using JASP v 0.95.3 (Amsterdam, The Netherlands) [28]. Nonparametric statistics were applied to compare variables between study groups, using the Mann–Whitney U test. Genotype analysis was performed using contingency tables and chi-square tests with Yates’ correction. Spearman’s rank correlation coefficients were calculated for continuous numerical variables. The frequency of MN and abnormal cells is reported by group.

## 3. Results

### 3.1. Characteristics of the Study Population

The study population comprised 70 patients in the ECIG group, 24 in the CCU group, and 110 in the NS group.

No statistically significant differences were observed between the ECIG and CCU groups in age, sex, weight, or height. Both groups began smoking at age 18. No differences were observed in the percentage of friends or parents who smoked, and no significant differences were observed in the pulmonary function test results. The FTND was fivefold higher in the ECIG group than the CCU group (5.5 vs. 1, *p* = 0.023), indicating nicotine dependence. Additionally, salivary cotinine levels were significantly higher in the ECIG group compared to the CCU group (Table 1).

The NS group consisted of 110 participants with a mean age of 21 years; 14.3% were male and had a body mass index of 23.1 kg/m^2^. They showed no abnormalities in the pulmonary function test, and their salivary cotinine level was zero. The FTND test was not performed, as this group consisted of non-smokers and non-e-cigarette users.

### 3.2. Characteristics Related to Tobacco Consumption

In the ECIG group, 72.85% (*n* = 51) were users of e-cigs and combustible cigarettes, consuming a median of 2 cigarettes per day, while the CCU group reported a median of 1 cigarette per day. The ECIG group reported a median of 2 years of vaping experience, with participants using the device 4.5 days per week and a median of 12.5 times per day. The average vaping session lasted 10 min, and the median inhalation time was 38 s.

**Figure 1:**
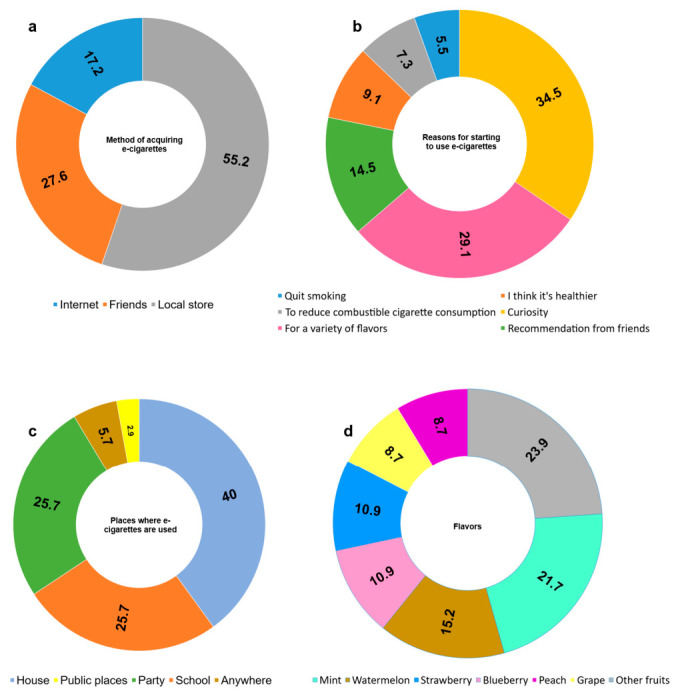
Description of the consumption pattern in dual users of e-cigarettes. (**a**) Method of acquiring electronic cigarettes. (**b**) Reasons for starting to use e-cigarettes. (**c**) Places where e-cigarettes were used. (**d**) The most popular flavors among e-cigarette users. Percentages are shown.

The most common source for acquiring e-cigarettes was a local store (55.2%), followed by friends (27.6%) (Figure 1a). Reasons for starting to use e-cigarettes included curiosity (34.5%), the variety of flavors (29.1%), and a friend’s recommendation (14.5%) (Figure 1b). The most common places for using e-cigarettes were participants’ homes (40%), followed by school (25.7%) or a party (25.7%) (Figure 1c). The most popular flavors among e-cigarette users were mint (21.7%), watermelon (15.2%), strawberry (10.9%), blueberry (10.9%), peach (8.7%), and grape (8.7%) (Figure 1d).

**Figure 2:**
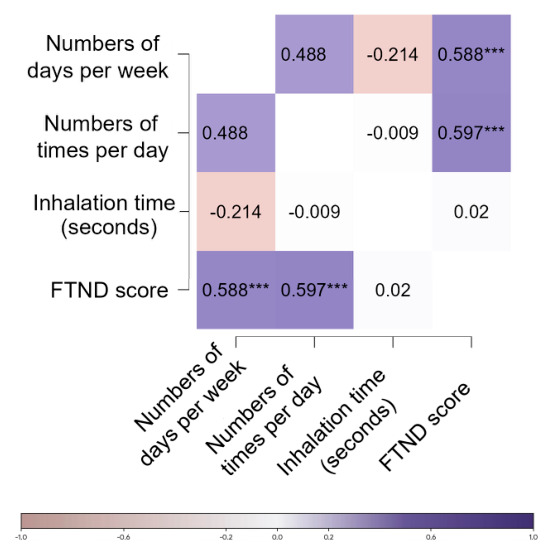
Spearman correlation heat map in the ECIG group, showing the number of times the device is used per week and per day, the approximate duration of an inhalation (seconds), and the score on the Fagerström Test for Nicotine Dependence (FTND). *** *p* < 0.001.

In the ECIG group, we observed a moderate and significant correlation (*p* < 0.001) between the FTND score and the number of days per week the electronic cigarette was used, as well as the number of times per day the device was used (rho = 0.588 and 0.597, respectively) (Figure 2).

### 3.3. Genetic Association Analysis

The variants rs16969968 (*CHRNA5*), rs6313 and rs6311 (*HTR2A*), and rs4005144 (*CYP2A6*) did not show significant differences across the three groups analyzed. The rs1137115 (*CYP2A6*) and rs1800955 (*DRD4*) variants exhibited significant differences in genotype frequency between the ECIG and CCU groups (Table S3). In the ECIG group, stratification was based on the presence of the risk allele and was analyzed according to the pattern associated with e-cigarette use.

We used a recessive model and found that subjects carrying the A allele (GA or AA) at rs16969968 on *CHRNA5* had higher FTND scores (6 versus 2 in GG carriers, *p* = 0.018). For rs1800955 (*DRD4*), daily device use was higher among C-allele carriers (TC or CC) (15 versus six in TT carriers, *p* = 0.014). Finally, carriers of the T allele (TT or CT) in rs4105144 (*CYP2A6*) consumed e-cigarettes at a younger age (18 years vs. 19 years in CC carriers, *p* = 0.016), as shown in Table 2.

### 3.4. Micronucleus Analysis

**Figure 3:**
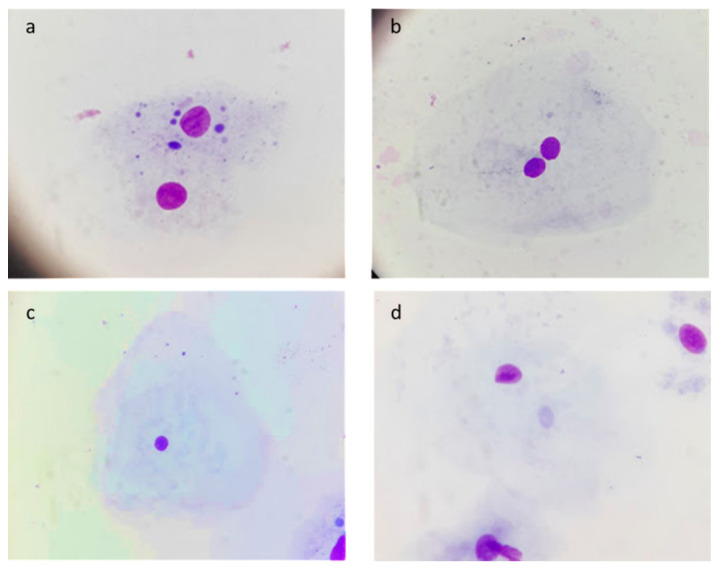
Micronuclei and nuclear abnormalities in the buccal epithelial cells of the ECIG group. (**a**) Cell with micronuclei. (**b**) Binucleated cell. (**c**) Cell with pyknosis. (**d**) Cell with karyolysis. The dark blue to purple color indicates the presence of chromatin.

Epithelial cells were obtained from a subgroup of participants who agreed to donate samples for MN analysis (Table 3). The NS group (*n* = 11) had low rates of nuclear abnormalities (micronuclei, binucleated, pyknosis, karyolysis) in their buccal epithelial cells; these rates were statistically significant compared with the ECIG group (Table 3 and Figure 3).

**Figure 4:**
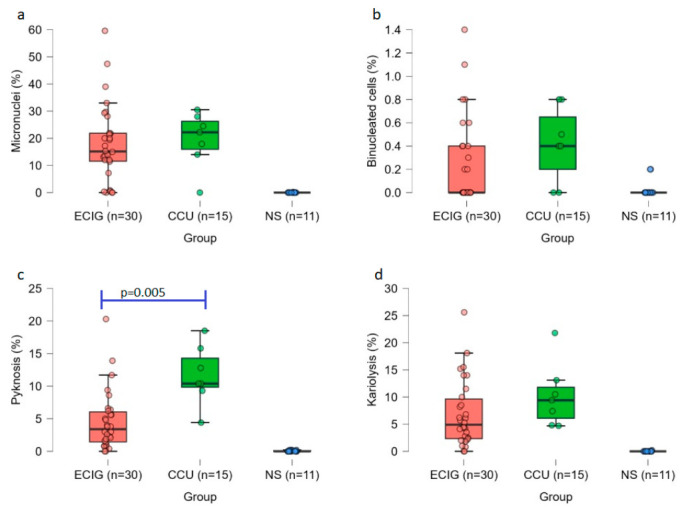
Box and whisker plots of micronuclei and nuclear abnormalities in the buccal epithelial cells of the study group. (**a**) Micronuclei. (**b**) Binucleated. (**c**) Pyknosis. (**d**) Karyolysis.

When comparing the ECIG (*n* = 30) and CCU (*n* = 15) groups, significant differences were observed in pyknosis (*p* = 0.005); MN counts, binucleated cells, and karyolysis did not differ significantly (Table 3). We observed greater dispersion in the percentage of cells with micronuclei and nuclear abnormalities in the ECIG group than in the CCU or NS groups (Figure 4). Among nicotine users, there was a significant difference in the percentage of pyknosis; however, compared with the NS group, there was a difference in all four cell morphological changes (Table 3).

## 4. Discussion

The use of electronic cigarettes is a public health problem that primarily affects adolescents and young adults [29]. There is evidence to suggest that the use of electronic cigarettes is a risk factor for starting to smoke combustible cigarettes [30]. This study focused on young adult university students who used both electronic cigarettes and combustible cigarettes. E-cigarette users scored higher on the FTND and had higher levels of cotinine in their saliva when compared to combustible cigarette users. Although cotinine is a widely accepted biomarker for monitoring nicotine consumption, the cutoff values for defining smoking status vary depending on the population studied [31]. When comparing salivary cotinine levels in our population, we observed that Caucasian participants had ranges from 100 to 300 ng/mL; however, these values cannot be generalized. It has been shown that the serum cutoff values for defining a smoker’s status vary according to race/ethnicity. Among non-Hispanic Black individuals, the cutoff is 2.77 ng/mL; for non-Hispanic White individuals, the values are 2.95 ng/mL, and 1.18 ng/mL for non-Hispanic White and Mexican Americans, respectively [32].

We also evaluated genetic variants previously associated with phenotypes related to nicotine addiction [33,34]. We found no significant differences between the genotypes of the ECIG and CCU groups; however, in the ECIG group, the A (rs16969968) and C (rs1800955) alleles in the *CHRNA5* and *DRD4* genes were associated with higher FTND scores or increased daily device use. The presence of the A allele (rs16969968/*CHRNA5*) has been widely associated with an increased risk of nicotine addiction in Caucasian, Asian, African American, and Latin American populations [35]. In addition to its role in nicotine addiction, in vitro studies have shown that this allele encodes a protein that leads to remodeling and a deficient immune response in the airway epithelium [36]. Following chronic nicotine use, the presence of the A allele is a risk factor that contributes to the development of lung disease [37].

Conversely, the T (rs4105144) allele in *CYP2A6* is associated with earlier ages of e-cigarette use. The CYP2A6 pathway is involved in nicotine metabolism. In the Caucasian population, rs4105144 is in high linkage disequilibrium with rs1801272 (CYP2A6*2), a reduced-function allele [12,38].

In our study population, based on the reported levels of e-cig or combustible cigarette use, no impairment of pulmonary function was observed. However, genotoxic and cytotoxic damage to the oral mucosa of these users was significantly increased compared to those who never used nicotine products. There were no statistically significant differences in genotoxic damage between dual users and combustible cigarette users. Similar findings have been previously reported regarding the amount of MN in populations from the United Arab Emirates and Romania, where groups of exclusive e-cig users showed no significant differences compared to combustible cigarette smokers [25,39]. Micronuclei are structures that form after genomic damage in oral mucosal cells and could serve as indicators of oral or lung damage [40].

Our study has some limitations, including the inability to include an exclusive e-cig user group for comparison and a modest sample size. However, dual e-cigarette use poses comparable genotoxic risks to combustible smoking, and certain genetic polymorphisms may predispose individuals to heightened nicotine addiction regardless of the delivery system. Although the study population could be considered users or light smokers, the findings obtained show similar levels of harm regardless of the form of nicotine consumption.

## 5. Conclusions

These findings suggest that dual e-cigarette use poses comparable genotoxic risks to combustible smoking. The alleles A (rs16969968), C (rs1800955), and T (rs4105144) were identified in the *CHRNA5*, *DRD4*, and *CYP2A6* genes, respectively, in the e-cigarette group; these alleles are associated with greater nicotine dependence.

## Figures and Tables

**Table 1:** Demographic data of dual and combustible cigarette smokers in the study.

Variable	ECIG (*n* = 70)	CCU (*n* = 24)	*p*-Value
Age (years)	20 (19–21)	20 (19–22)	0.592
Sex, Male (%)	30	43	0.836
BMI (kg/m^2^)	23.4 (21.7–25.1)	24.5 (21.7–25.7)	0.479
Age at onset (years)	18 (17–20)	18 (16–18)	0.377
Smoking friends, %	83.4	71.4	0.850
Smoking parents, %	73.3	42.9	0.270
FVC%	100.5 (93.2–108.0)	94.5 (90.0–96.5)	0.276
FEV1%	97 (90–107.5)	91 (86.7–93.2)	0.121
FEV1/FVC	98 (94–101)	103 (99–106)	0.150
Modified FTND	5.5 (1.75–9.00)	1 (1–2)	0.023
Cotinine in saliva (ng/mL)	39 (11–62)	12 (0–32)	<0.001

We show the median (25–75 percentile). *p*-value by Mann–Whitney U test. FVC: Forced Vital Capacity; FEV1: Forced Expiratory Volume in the first second; ECIG: e-cigarette dual users; CCU: exclusive users of combustible cigarettes; FTND: Fagerström Test for Nicotine Dependence [26].

**Table 2:** Stratified analysis of risk alleles in the studied genes and variables related to smoking in the ECIG group.

SNP/Gene	Genotype		*p*-Value
rs16969968/*CHRNA5* (*n* = 66)	GG (*n* = 49)	GA or AA (*n* = 17)	
FTND	2 (1–8)	6 (5–10)	0.018
rs1800955/*DRD4* (*n* = 67)	TT (*n* = 28)	TC or CC (*n* = 39)	
Device usage times per day	6 (3–10)	15 (4–30)	0.014
rs4105144/*CYP2A6* (*n* = 70)	CC (*n* = 29)	CT or TT (*n* = 41)	
Age at onset (years)	19 (17–20)	18 (17–19)	0.016

Median (25–75 percentile) shown; *p*-value by Mann–Whitney U test. ECIG: e-cigarette dual users; FTND: Fagerström Test for Nicotine Dependence.

**Table 3:** Percentage of MN and nuclear abnormalities in the buccal epithelial cells of the study population.

Nuclear Abnormalities (%)	NS (*n* = 11)	ECIG (*n* = 30)	*p*-Value *	CCU (*n* = 15)	*p*-Value **
Micronuclei	0 (0–0)	15.5 (11.5–21.8)	<0.001	22.2 (15.9–26.2)	0.725
Binucleated	0 (0–0)	0 (0–0)	0.003	0.4 (0.2–0.6)	0.248
Pyknosis	0 (0–0.05)	3.4 (1.4–6.0)	<0.001	10.4 (9.8–14.3)	0.005
Kariolysis	0 (0–0	4.9 (2.3–9.6)	<0.001	9.4 (6.1–11.8)	0.213

Median (25–75 percentile) shown. NS: non-smokers and non-users of e-cig; ECIG: e-cigarette dual users; CCU: exclusive users of combustible cigarettes. * *p*-value by Mann–Whitney U test between NSs and ECIGs. ** *p*-value by Mann–Whitney U test between ECIGs and CCUs.

## Data Availability

The data presented in this study are openly available in https://www.ncbi.nlm.nih.gov/clinvar/?term=%22HLA%20Laboratory%2C%20Instituto%20Nacional%20de%20Enfermedades%20Respiratorias%20Ismael%20Cosio%20Villegas%22%5bsubmitter%5d (accessed on 10 February 2026).

## Supplementary Materials

The following supporting information can be downloaded at https://www.mdpi.com/article/10.3390/arm94030039/s1: Table S1: Characteristics of SNPs and assays employed. Table S2: Preparation of Sorensen’s phosphate buffer pH 7. Table S3: Genotypic and allelic frequencies of selected SNPs.

## Abbreviations

The following abbreviations are used in this manuscript:

e-cig	e-cigarette
SNPs	Single-nucleotide polymorphisms
MN	Micronucleus
ECIG	Dual e-cig users
CCU	Users of combustible cigarettes
NS	Non-smokers and non-users of e-cigs
INER	Instituto Nacional de Enfermedades Respiratorias Ismael Cosío Villegas
PCR	Polymerase Chain Reaction
ELISA	Enzyme-linked immunosorbent assay
