# Supplementary material for: Dual E-Cigarette Users Show Nicotine Addiction Risk Alleles and Nuclear Abnormalities in Oral Epithelial Cells
Source: Adv Respir Med. 2026 Jun 18;94(3):39. doi: 10.3390/arm94030039 (PMC13295726; doi:10.3390/arm94030039)
Supplement: Supplementary file 1 [file arm-94-00039-s001.zip › arm-4273560-supplementary.pdf]

Table S1. Characteristics of SNPs and assays employed.

SNP	Taqman Assay ID	Allele	Gene
rs16969968	C__26000428_20	A/G	*CHRNA5*
rs6313	C___3042197_1_	A/G	*HTR2A*
rs6311	C___8695278_10	C/T	
rs1800955	C___7470700_30	C/T	*DRD4*
rs1137115	C__26681694_20	C/T	*CYP2A6*
rs4105144	C__43815914_20	T/C	

Table S2. Preparation of Sorensen's phosphate buffer pH 7

Substance	Quantity
Distilled water	as much as is enough to 1,000 mL
KH_2PO_4	6.63 g (Sigma-Aldrich, Darmstadt, Germany)
Na_2HPO_4	2.56 g (Sigma-Aldrich, Darmstadt, Germany)

Table S3. Genotypic and allelic frequencies of selected SNPs.

rs16969968	ECIG (n=70)		CCU (n=24)		NS (n=110)		p-value*
CHRNA5	n	%	n	%	n	%	
GG	53	75.7	15	62.5	80	72.7	0.404
GA	16	22.9	8	33.3	30	27.3	
AA	1	1.4	1	4.2	0	0.0	
G	122	87.1	38	79.2	190	86.4	0.269
A	18	12.9	10	20.8	30	13.6	
rs6313							
HTR2A	n	%	n	%	n	%	
GG	36	51.4	17	68.0	80	72.7	0.348
GA	31	44.3	7	28.0	30	27.3	
AA	3	4.3	1	4.0	0	0.0	
G	103	73.6	41	82.0	190	86.4	0.316
A	37	26.4	9	18.0	30	13.6	
rs6311							
HTR2A	n	%	n	%	n	%	
CC	33	47.1	10	40.0	70	63.6	0.845
CT	34	48.6	14	56.0	40	36.4	
TT	3	4.3	1	4.0	0	0.0	
C	100	71.4	34	68.0	180	81.8	0.782
T	40	28.6	16	32.0	40	18.2	
rs1800955							
DRD4	n	%	n	%	n	%	
TT	29	41.4	11	44.0	110	100.0	**0.016**
TC	34	48.6	6	24.0	0	0.0	

CC	7	10.0	8	32.0	0	0.0	
T	92	65.7	28	56.0	110	100.0	0.164
C	48	34.3	22	44.0	0	0.0	
rs1137115							
CYP2A6	n	%	n	%	N	%	
CC	42	60.0	15	60.0	80	72.7	
CT	20	28.6	2	8.0	20	18.2	**0.019**
TT	8	11.4	8	32.0	10	9.1	
C	104	74.3	32	64.0	180	81.8	0.620
T	36	25.7	18	36.0	40	18.2	
rs4105144							
CYP2A6	n	%	n	%	n	%	
TT	4	5.7	2	8.3	30	27.3	
CT	59	84.3	19	79.2	60	54.5	0.839
CC	7	10.0	3	12.5	20	18.2	
T	67	47.9	23	47.9	120	54.5	0.872
C	73	52.1	25	52.1	100	45.5	
